# Factors influencing well-being among first-generation students of color pursuing graduate-level degrees in public health: A qualitative study^☆^

**DOI:** 10.1016/j.dialog.2025.100242

**Published:** 2025-09-28

**Authors:** Kimberly Wu, Sunshine Best, W. Marcus Lambert, Shokufeh Ramirez, Christine M. Arcari, Katherine P. Theall, Dovile Vilda

**Affiliations:** a Department of Social, Behavioral, and Population Sciences, Celia Scott Weatherhead School of Public Health and Tropical Medicine, New Orleans, LA 70112, United States of America; b SUNY Downstate Health Sciences University, Brooklyn, NY 11203, United States of America; c Department of Epidemiology, Celia Scott Weatherhead School of Public Health and Tropical Medicine, New Orleans, LA 70112, United States of America

**Keywords:** Public health training, Workforce development, Equity

## Abstract

Diversifying the public health workforce is essential for improving services to all communities. First-generation students of color (FGSOC) are a population who may face unique academic, financial, and health barriers when pursuing graduate-level degrees in public health. This study explored the needs and experiences of 25 recent FGSOC graduates from graduate-level public health programs through interviews. Participants were recruited through snowball and quota sampling to prioritize individuals identifying as Black, Hispanic/Latine, and American Indian/Native Alaskan. Five main themes were developed using reflexive thematic analysis: (1) lived experiences facilitate FGSOC interest in public health, (2) challenges of navigating structural barriers of academic institutions, (3) interplay of support to meet needs and complete graduate degrees, (4) impact of limited resources on health, and (5) coping strategies for maintaining health. Subthemes were also identified and include participants' firsthand experience with health disparities in their families and communities, the additional effort needed to uncover and navigate academic “hidden curricula,” and the importance of cultivating a network to meet emotional and academic needs during their training. Findings highlight how FGSOC are driven by formative experiences to pursue public health, yet face structural barriers and multiple priorities that strain their well-being during their graduate training. Recommendations informed by study findings and existing literature are organized across the following domains: institutional accountability and investment, financial education and transparency, career development and workforce readiness, accessible mental health resources, and food security.

## 1 Introduction

While enrollment of racially and ethnically underrepresented students in undergraduate and graduate-level public health programs has increased in recent years, demographic representation is still not reflective of the diversity of the U.S population [1]. A diverse public health workforce enhances systemic capacity to develop culturally responsive programs and policies, strengthening the health infrastructure to better serve all communities [[2], [3], [4], [5], [6], [7]]. A longitudinal study using 2016–2020 data from the Association of Schools and Programs of Public Health (ASPPH) created an institutional diversity index (DI) to assess the demographic background of public health graduates to the diversity of the population in the geographic area where schools are located [1]. Findings from the study highlighted underrepresentation of students from racially and ethnically minoritized groups, such as Black, Hispanic, American Indian/Alaska Native, and Native Hawaiian/Pacific Islander populations in public health programs. As a result, the authors emphasized the need for additional research and data to account for the nuanced experiences and outcomes of diverse public health students, particularly by including measures of class and first-generation (FG) status [1].

The unique undergraduate experiences of FG students are widely studied and documented in existing literature, primarily related to academic performance [8], mental health [9,10], familial relationships [11,12], and identities [13,14]. However, fewer studies examine the experiences of FG students pursuing graduate degrees [15], and even less research exists on the health outcomes and experiences of FG graduate students pursuing public health degrees. About 3 % of FG college graduates go on to pursue and complete graduate or professional programs, and academic institutions should be prepared to understand and serve the needs of this population [16]. While FG graduate students may have successfully completed their undergraduate degrees, institutions cannot assume the transferability of past academic experiences to the skills and knowledge needed to navigate graduate programs.

In fact, recent studies highlight the mental health crisis among graduate students [[17], [18], [19], [20], [21]]. Research into the well-being of graduate students in other healthcare fields have been documented for medicine [22], dentistry, [23], physical therapy [23], nursing [24], and pharmacy programs [25]. Graduate students of color also face challenges related to racism, which not only create barriers in navigating systems of higher education, but can also adversely impact health outcomes [26,27]. To address this gap in the literature, our study was designed to prioritize the health and public health graduate training experiences of first-generation students of color (FGSOC). FGSOC is defined as individuals who exist at the intersection of identifying as both FG students and racial minorities [28]. We aimed to examine the needs of FGSOC by drawing on the perspectives of self-identified recent graduates from master's and doctoral public health programs from across the United States. This study is guided by the following questions:

1. What factors impact FGSOC's ability to complete graduate-level public health degrees?
2. What are FGSOC's experiences with their mental, physical, and emotional health and well-being during their public health graduate training?
3. What recommendations emerge from the study to inform changes to policy and practices for public health graduate-level programs and schools?

To facilitate our inquiry into the public health training experiences of recent FGSOC, we draw on conservation of resources and intersectionality theories. Hobfoll's conservation of resources (COR) theory provides a framework for conceptualizing the relationship of resources within the context of graduate training experiences for FGSOC. According to Hobfoll, resources are “loosely defined as objects, states, conditions, and other things that people value” (p.2) that could affect coping strategies when faced with stressors [29]. COR theory has been previously used in research on student burnout and engagement with first-year college students to identify the impact of resources on managing stress [30].

Our study focuses on the roles that resources – personal (aspect of one's personality or beliefs), social (types of social support), and economic (income, other material means) – play on FGSOC's health and graduate training experiences [31]. Furthermore, to account for greater nuance in diversity, we adopt an intersectional approach to consider the interrelated relationships of power, privilege, and oppression when analyzing the experiences of FGSOC within U.S higher education systems [32]. Originating from the legal scholarship of Kimberlé Crenshaw, intersectionality theory allows researchers to explore the complexity of the interlocking effects of macro-level oppression on individuals' [33,34]. Crenshaw's work recognized how the unique experiences of individuals existing at the intersections of multiple marginalized identities are often overlooked [34]. In the context of this research, intersectionality provides a framework for examining the distinct needs and health outcomes of FGSOC in public health. Taken together, our theoretical approach links access to resources during public health graduate training experiences of FGSOC to the historical and structural dynamics of power that perpetuate systemic injustices.

## 2 Methods

This qualitative study was part of a larger project that aimed to examine graduate education and transition to workforce experiences of recent FG graduates of color in the field of public health. The current study outlined below utilized data collected from 25 semi-structured in-depth interviews with FG public health graduates of color. This study was approved by the Tulane University Health Sciences Center Institutional Review Board. Furthermore, a completed Consolidated Criteria for Reporting Qualitative Research (COREQ) checklist is provided in Supplementary Material.

Eligibility criteria for study participants included identifying as a FGSOC who completed graduate level training in public health within the last 10 years. For this study, FG students were defined as individuals who completed a four-year college degree whose parents had not attained higher education. To ensure adequate representation from underrepresented subgroups, individuals were recruited from the following intersectional identity groupings: (1) Black, FG, female; (2) Black, FG, male; (3) Hispanic/Latinx, FG, female; (4) Hispanic/Latinx, FG, male; (5) American Indian/Native Alaskan, FG, female; and (6) American Indian/Native Alaskan, FG, male). The decision to focus on these specific intersections of identity was based on the evidence that Black, Hispanic/Latinx, and American Indian/Native Alaskan students make up a higher percentage of FG students than Asian and White students [35]. Due to challenges in recruiting Black, FG, male participants, the sample was expanded to include interviews with Asian and Multiracial, FG, males.

Participants were recruited through snowball and quota sampling. Individuals from a parent study on the career experiences of recent public health graduates from graduate-level training programs were invited to participate. Prospective participants were screened using a Qualtrics survey to collect basic information, confirm eligibility, and indicate availability for an interview. Eligible participants were contacted via email in March 2024 with a study description and encouraged to share the study with other eligible individuals. Quota sampling prioritized individuals at key identity intersections (i.e., race/ethnicity, education generation status, and gender) [36]. The study sample was not representative of the parent study, as it focused on individuals underrepresented in public health. Recruitment materials were also shared through social media and professional listservs (electronic mailing lists used to distribute information to groups of subscribers or members).

Semi-structured interviews were conducted using Zoom with participants' consent, including permission to record for transcription. A total of 25 participants were interviewed between April 10, 2024, and June 25, 2024. Anonymity and confidentiality were ensured by masking participants' names and password protecting files. No identifying information was included in the final qualitative dataset, analysis, or final write up of the study's findings. Participants received a $25 Amazon digital gift card for their participation in the study. The interview guide, developed and piloted with three FGSOC, was revised based on feedback to improve question logic, flow, and interpretability for study participants. The interview guide included ten open-ended questions related to participant's higher education experiences, health during graduate training, and the roles of personal, social, and economic resources. Virtual interviews lasted on average one hour, ranging from 35 to 90 min. The research team did not establish relationships with participants prior to the study.

Interview data was analyzed following Braun and Clarke's six-phase reflexive thematic analysis framework [37]. Audio recordings were transcribed using Zoom then deidentified and verified for accuracy by two members of the research team. Transcripts were not returned to participants for their feedback. Initial codes were generated using the interview guide as a coding framework in Atlas.ti. Code and meaning saturation were assessed based on the recurrence of main themes and sub-themes, with no new codes or meaning emerging from additional interview analyses. Previous research suggests that code saturation can be achieved with 9 interviews, and meaning saturation with 16–24 interviews [38]. Two research team members conducted coding using an iterative process, revising previous codes while moving between transcripts. Both deductive and inductive approaches were employed, with deductive analysis framed by the interview guide, and inductive analysis without any predetermined frame emerging from participants' responses [37]. Semantic analysis summarizing existing data, and latent analysis requiring the researchers' active role in interpreting underlying ideas and meaning were also adopted [37,39,40]. Codes were consolidated in a shared spreadsheet and key themes and subthemes identified. Member checking was not adopted due to time and resource limitations.

The research team's positionalities reflect their intersecting identities, lived experiences, and training as doctoral-level public health students (DrPH and PhD) with qualitative research experience, conducted under the guidance of faculty with extensive background in qualitative methods. One member, a cis-woman, Afro-Caribbean-Canadian immigrant from a low-income background with high Adverse Childhood Experiences (ACES), brings insight into health disparities and social determinants of health. The other, a FG student, Asian American, cis-woman, and child of immigrants, contributes perspectives shaped by her experiences in education and social justice work.

## 3 Results

From Table 1, of the 25 study participants, 12 (48 %) individuals identified as female, 12 (48 %) individuals identified as male, and one individual identified as non-binary. Twenty-two (88 %) of the participants spoke about their experiences completing a master's-level degree in public health and three participants (12 %) reflected on doctorate level degrees- one Doctor of Public Health (DrPH) and two for Doctor of Philosophy (PhD) in Public Health. Eleven (44 %) completed their graduate level public health degrees in private academic institutions while 14 (56 %) completed their degrees in public academic institutions. Approximately one-third of the sample were geographically based in the Northeast, although participants hailed from across the United States.

**Table 1:** Demographic overview of study sample.

Demographic Characteristics		Number of Respondents (*N* = 25)	Percentage of sample
Gender	Female	12	48 %
	Male	12	48 %
	Non-Binary	1	4 %
Race	Asian	1	4 %
	Black	6	24 %
	Latinx	13	52 %
	Native American/AI	3	12 %
	Multiracial	1	4 %
Graduate Degree Type	MPH	21	84 %
	MS	2	8 %
	PhD	2	8 %
	DrPH	1	4 %
Institution Type	Public	14	56 %
	Private	11	44 %
Geographic Region	Northeast	8	32 %
	MidAtlantic	1	4 %
	Southeast	1	4 %
	South Central	2	8 %
	Southwest	1	4 %
	Midwest	3	12 %
	Northwest	2	8 %
	West	4	16 %

From the analysis, five themes and 13 subthemes were identified from the interview transcripts in relation to the research questions addressed in this study. As illustrated in Table 2, themes included (1) lived experiences facilitate FGSOC interest in public health, (2) challenges of navigating structural barriers of academic institutions, (3) interplay of support to meet needs and complete graduate degrees, (4) impact of limited resources on health, and (5) coping strategies for maintaining health*.*

**Table 2:** Overview of study themes and subthemes.

Theme	Subtheme
1. Lived Experiences Facilitate FGSOC Interest in Public Health	• Firsthand experience of health disparities in their families and communities • Sense of kinship and obligation for equity in public health
2. Challenges of Navigating Structural Barriers of Academic Institutions	• Extra effort needed to uncover “hidden curricula” and navigate cultural disconnect • Balancing financial realities with education/training
3. Interplay of Support to Meet Needs and Complete Graduate Education	• Strategies for navigating systems of higher education • Identifying a network for emotional support and academic needs
	• Engaged mentors as trusted guides of higher education systems
4. Resource Strains as Stressors	• Limited time • Pressure to keep costs/debts low • Pressures not to fail
5. Coping Strategies for Well-being	• Benefits of the sense of belonging
	• Accessibility to health promoting resources
	• Alleviating financial burdens reduces stress

### 3.1 Theme 1: lived experiences facilitate FGSOC interest in public health

Participants described multiple factors motivating their decisions to pursue public health graduate training. Many discussed how firsthand experiences with health disparities shaped their understandings of the connections to structural disadvantage. These experiences fostered a sense of responsibility among participants to give back to their communities and highlighted their lived experiences as insider knowledge for serving their communities effectively.

#### 3.1.1 Subtheme 1.1: firsthand experience of health disparities in their families and communities

Of the 25 participants, most (*n* = 15) recognized the importance of lived experiences in shaping their decisions to pursue a public health degree and career. Many described growing up in low socio-economic backgrounds, where they observed inequities affecting marginalized communities' education and preparedness for disease prevention and management. These experiences fostered personal connections to systemic disadvantages and greater awareness of the gaps in addressing health disparities.

*“For me being a first-generation public health graduate student has shown me that, when not enough conversation [is] being had about certain health topics, especially in communities that have historically been disadvantaged or marginalized, it is harder for people to get the knowledge and the resources that they need so they can live a healthy lifestyle.”* – P07 (Black, Female).

Participants also reflected on childhood experiences of facing barriers to healthcare, often through the challenges faced by relatives (parents, grandparents, aunts) and other community members. A common theme was that lived experiences motivated them to pursue public health as a way of addressing these gaps. One participant, who grew up in migrant camps, recalled: “*Growing up I would always live in migrant camps, and the only time that we would have access to a doctor, or any health-related resources was when the health workers would come to us…I just want to do something about it. It shouldn't have to take someone's volunteer hours for me to get healthcare.”* —P24, Latinx, Male. This quote speaks to the intimate knowledge FGSOC have of experiencing health inequities, and how these experiences drive their commitment to improve public health.

#### 3.1.2 Subtheme 1.2: sense of kinship and obligation for equity in public health

As FGSOC described the importance of their lived experiences and identities as motivation to pursue graduate-level training in public health, many emphasized the sense of accountability to marginalized communities. Participants linked their personal experiences with accessing healthcare to a commitment to prevent similar struggles for others. One participant explained:

*“My passion for helping others has really helped a lot. I think about the future generations, and it motivates me so much because I don't ever want someone to go through what I've gone through. I want them to have someone to lean on, whether that's a trusted adult, a peer, a mental health professional. I don't want people to be scared.” –* P09 (Latinx, Female)

These formative childhood experiences were integral to shaping participants' decisions to pursue public health. Thirteen participants highlighted this sense of kinship with communities of shared or marginalized identities, often describing it as a connection to a broader group. These early life experiences fostered empathy and strengthened a commitment to address health inequities in their training.

### 3.2 Theme 2: challenge of navigating structural barriers of academic institutions

Another theme that arose was the challenge FGSOC faced navigating systems of higher education, including uncovering “hidden curricula” and confronting power imbalances rooted in of cultural and socio-economic inequities. “Hidden curricula”, or the “unspoken or implicit values, behaviors, and norms that exist in the educational setting” [41], reflect the cultural, social, and academic knowledge gaps that FGSOC must confront. Nine participants described feeling marginalized and the impact of that marginalization on their studies, identities, and relationships within their academic institutions.

#### 3.2.1 Subtheme 2.1: extra effort needed to uncover “hidden curricula” and navigate cultural disconnect

Nine (36 %) participants alluded to the extra labor required to identify gaps in knowledge necessary for navigating systems of higher education while completing their training. These gaps were linked to their FG status and lack of familial experiences with graduate education. Participants often described themselves as trailblazers, creating new patterns and legacies for their families, without access to experiential knowledge or guidance. One participant recalled:

*“My family tried to support me as best as they could, but the ‘hidden curriculum’ existed in a lot of ways that I hadn't fully understood until I was in the midst of it. I realized that people were getting resources either through older family members or folks who have gone through the process that helped them to get to where they are.” –* P17 (Black, Female)

While participants had siblings, younger relatives, or close friends to lean on while completing their 4-year undergraduate degrees, they felt more alone when pursuing their graduate studies since fewer friends and relatives embarked on that path. Beyond navigating knowledge gaps, participants confronted cultural and class barriers during their studies. They described feelings of being “othered” and disconnect between their lived experiences with course content. This awareness of the epistemic differences negatively influenced perceptions of academic institution. One participant described her experience:

*“A common theme in my public health training was the lack of thinking about things that are really important. Social determinants of health are super important, and we should care about communities, but are we really doing that in the most appropriate way? My lived experiences as [a] Brown Latina in the Southern US influence those things. Other people's lack of understanding and making decisions about curriculums and policies [need] to take these into consideration”.* P15 (Latinx, Female)

This quote underscores the awareness and challenges FGSOC face in navigating power dynamics and cultural codes embedded in higher education that other students may not have to confront. It also raises FGOSCs' ability to identify gaps in public health training, especially around working effectively with diverse communities. One participant emphasized the value of having mentors of diverse backgrounds to navigate hidden curricula and promote self-advocacy:

“*When I was applying for my master's program, someone told me, you should try to negotiate your financial aid. I did it, and it worked in my favor. Those are things that were never told to me- that you can advocate for yourself in this way. We don't know to ask for them. And that also goes to having more diverse mentors who can give first-gen students and other students of color this institutional knowledge that nobody's going to tell you.’”*—P11 (Latinx, Male)

#### 3.2.2 Subtheme 2.2: balancing financial realities with education/training

Another barrier identified by participants was balancing financial realities with academic and personal responsibilities. Nine participants described the challenges of completing unpaid practicum internships as part of fulfilling program requirements. This investment of tuition fees, time, and effort while leading to potential long-term future benefits often created short term financial stress. They highlighted the difficulty of managing financial alongside programmatic demands. One participant described his experience:

*“I was working for most of my grad program. Last week was very hard because I quit my other [paid] job because I wanted to fully concentrate on the program. Having a job and going to school- it's difficult with the workload. The way our program works is, in order to graduate, we do an internship with an organization that's similar to our concentration. I did mine in epidemiology, so I went to the Health Department. And obviously, this is not paid.”--* P24 (Latinx, Male)

Some participants noted the challenge of observing classmates receive financial support from their families that facilitated future career development. Experiences of short-term financial instability led participants to take out student loans or find other ways to financially support themselves. One participant reflected on the inequitable learning opportunities shaped by financial realities:

*“This one girl in my program, she was White, and we all knew that she came from a wealthy family. She did her practicum at the WHO in Geneva. And this is the practicum that's gonna be spotlighted in the newsletter. She doesn't have to worry about not being paid… (laughs) While I'm here in Tennessee, not being paid, worrying about my bills and stuff like that. So, yeah, that particular part of the master's experience was just incredibly frustrating and showed the lack of consideration for equity.”--* P15 (Latinx, Female)

On the other hand, participants identified tuition remission and the financial assistance while working for academic, government, and nonprofit organizations as a critical resource. However, such benefits required balancing the added workload and responsibilities of a full-time or part-time job alongside their graduate training. These results highlight the importance of recognizing the effort FGSOC invest in to maintain financial stability during their training experiences.

### 3.3 Theme 3: interplay of support to meet needs and complete graduate education

Many participants identified strategies for coping with barriers, which included employing creative ways to offset costs, building networks to meet emotional and academic needs, and drawing on trusted mentors for academic and practical counsel. We identified three subthemes highlighting coping strategies that strengthened participants' personal, social, and economic resources.

#### 3.3.1 Subtheme 3.1: strategies for navigating systems of higher education

Eight (32 %) participants discussed strategies to reduce financial burdens during their graduate training, including applying for scholarships, working full-time for tuition remission benefits, and sharing resources such as textbooks and course-related information with classmates and friends. One participant described leveraging the bonds of a small cohort, *“We would do study sessions at different people's apartments. It was a good chance for folks to save money. If everyone brought one or two snacks, you basically had a full meal (laughs). We found ways to get the school to sponsor dinner or like pizza party studying together.”* —P17 (Black, Female).

For many participants, peer and friend support was critical in expanding access to tangible financial support. One participant described how friends provided food, gas money, and even co-signed a loan to help him continue his education: *“There was the time I gave up…am I going to finish this thing? … one of them actually co-signed for me to encourage me to go through with my education, otherwise I would have dropped out.”* —P22 (Black, Male).

#### 3.3.2 Subtheme 3.2: identifying a network for emotional support and academic needs

Eighteen (72 %) participants emphasized the importance of cultivating a network for emotional and academic support during their graduate training. While participants found emotional support from classmates, family, and significant others, peers played particularly important roles in filling gaps that professors or family members could not meet. Participants felt more comfortable approaching their peers than faculty for academic questions, especially when bonds of friendships were strong. As one participant described, “*It gets a little difficult when you try to talk to family members, especially being a first-generation student, because they're not familiar with that kind of stress [and] challenges.*” —P21 (Native American, Female).

In addition to academic support, peers and family members motivated participants to persist in completing their training and overcome obstacles in their path. One participant reflected on the value of peer connections with members of his cohort: “*I know I'm supposed to reach out to the professors, but (laughs) I was just scared to. I found that difficult. I know I should have, but I didn't. I reached out to a friend who was able to help me with this program.”* —P24 (Latinx, Male). Lastly, student organizations created important connections of cultural familiarity, especially for first-generation students navigating structural barriers and instances of discrimination. One participant described witnessing institutional practices during the pandemic privileging student needs over the community and identified connecting with other first-generation students as a means of countering racism: *“The fight definitely solidified these strong bonds between me and other first-generation students trying to fight for representation in research.”* —P20 (Multiracial, Male).

#### 3.3.3 Subtheme 3.3: engaged mentors as trusted guides of higher education systems

Thirteen (52 %) participants highlighted the significant role of mentors in bridging aspects of the “hidden curricula” and offering counsel on critical future career decisions. Effective mentors were described as trustworthy, honest, and attentive to participants' realities, thereby building trust to provide practical and professional guidance about job options and careers directions. One participant recounted:

*“I've been really blessed to have people who filled that gap of not having parents who went to college. I was really able to rely on them for advice. Early on in my undergrad, from my first year, I had a good mentor who told me all about graduate school and PhDs. Because of that, I was able to solidify my goals and realize I had to do research throughout my undergrad and get publications.” –* P13 (Latinx, Male)

Mentors with shared identities and experiences provided additional support by connecting on values and cultural contexts while helping students navigate their graduate education. One participant recalled how a mentor of the same ethnic background offered deeper guidance and preparation during her doctorate training:

*“First time in my life I had a Latino [mentor], a person of my same ethnicity. He really took advantage of that time to mentor me to prepare me [for] beyond what the program was preparing me for. And part of that was seeing, she's the only Latina person in this program. And from that shared cultural identity, he [had] a vested interest in me, maybe even saw a little bit of his experience in what I was going through.”* – P15 (Latinx, Female)

### 3.4 Theme 4: resource strains as stressors

Our analysis also revealed that challenges managing threats to personal, social, and economic resources worsened existing health concerns or created new mental or physical health issues. Resource strains included limited time, pressures to keep costs low for financial stability, as well as pressures not to fail. We identified three subthemes highlighting conditions that strain resources and adversely impact participants' health during their graduate training.

#### 3.4.1 Subtheme 4.1: limited time

Over half of participants (*n* = 15, 60 %) raised health concerns due to limited time while managing multiple responsibilities. Demands from graduate coursework, combined with the stress of managing work and basic living needs, left many feeling overwhelmed and unbalanced. Participants reported increased sense of isolation, and in some cases, described clinical diagnoses of anxiety and depression. As one participant explained:

*“I hadn't quite figured out the perfect balance of managing school, work, and also taking a step back for myself to just breathe. I just felt overworked. Being first-generation to not only get the undergraduate degree, but now pursuing the master's degree, there's no one to talk to about the things that I'm going through, because nobody in my family has ever done it. And then on top of that, I feel this immense amount of pressure to just get it done, because I know my family will be super proud and because it's something that they've wanted for me since I was young. During my graduate degree, I definitely dealt with a lot of anxiety and depression, and now I still deal with it”--* P08 (Black, Female)

Several participants raised the changes to their habits and routines when completing their graduate training, particularly around sleep patterns, physical activity, and diet. Limited time often meant sacrificing rest and healthy behaviors. One participant reflected:

*“With my commute, I wasn't getting as many steps, I'm not getting enough sleep because I have to wake up the next morning for work. And then throughout that day, I'm not really eating healthy, and I'm not getting enough physical activity. I noticed that I had gain[ed] at least 50 pounds. It was just a lot of hours that I was committing to school and work. I just didn't have enough time for myself.”*—P07 (Black, Female)

As this quote demonstrates, participants described focusing their energy into completing their degrees, often at the cost of neglecting their own health and well-being. The stress of limited time is further compounded academic demands, jobs responsibilities, and the environmental realities of where they lived, leaving little room for maintaining healthier habits. One participant described: “*Physically, I just have my foot on the gas to just finish school. So, a lot of the things I probably ignored about myself.”--*P08 (Black, Female). These findings indicate that for many FGSOC, graduate training often came with experiencing the trade-off of physical and mental stress.

#### 3.4.2 Subtheme 4.2: pressure to keep costs/debts low

Most participants (*n* = 19, 76 %) reported working at least one full-time job, or multiple part-time positions to cover living and tuition costs. Many described earning just enough funds to maintain basic living expenses. One individual described his experience: “*It's not easy not having an income. I technically had three different jobs that when you sum it together was about 40 hours a week. And that helped, but it also made like the work week itself, with school, with volunteering, with all this other stuff, ended up being like 60 to 70 hours a week of being constantly being on, constantly working.”*--P20 (Multiracial, Male).

Several participants also described responsibilities of sending funds to relatives and the added stress that created. An international student described the tensions between these obligations and meeting academic goals: *“I was working two jobs and also going to school. I was stressed because even though I was a student, I still had to send money home to my family. Stress was high trying to figure out how to manage my own finances here, as well as supporting my family [while] trying to maintain that really high GPA to access scholarships or be on the dean's list.”--*P04 (Black, Female).

Participants also discussed the unexpected stressors during their graduate training, especially because of the COVID-19 pandemic. These included experiencing a loss in the family, as well as disruptions to job and internship opportunities leading to financial instability. One participant recounted providing financial support for relatives in the U.S who were unable to work during the pandemic. For some participants, financial pressures extended to future responsibilities. One participant spoke about experiencing chronic despair, depression, and anxiety knowing that his graduate training decisions had to account for providing financially for his parents, who were not U.S citizens nor had adequate retirement funds. Others described feelings of anxiety when comparing their situations to peers who were not in graduate school with time to build careers with more stable incomes.

For the participants who were eligible to take out student loans, the long-term impacts of these decisions were concerning. Some participants, specifically those with undocumented statuses, described barriers to accessing loans as an option. Those with student loan debt discussed the challenge of maintaining financial stability while anticipating future repayment, especially when balancing changing family responsibilities and partners' student loan debt. One participant described their experience as living in a state of financial fragility: *“Especially for first-gen people, lack of financial stability is something that is super triggering…we don't have those resources to fall back on. And that really hurt my mental health a lot.”*--P15 (Latinx, Female).

Several participants described accessing national scholarships from organizations that fund individual higher education opportunities, such as the Gates Foundation. Yet even with these resources, participants described challenges of managing daily living expenses, especially when attending schools in higher income urban areas. One individual described their experience: *“Even me with scholarships, it's been a struggle, so I can't imagine people who don't have that. And especially on top of that if you have debt, or your family's not supporting you, it can be a lot.”* —P13 (Latinx, Male).

#### 3.4.3 Subtheme 4.3: pressures not to fail

Twelve participants (48 %) described pressures not to fail resulting from both internal and external forces. For some, this sense stemmed from honoring the sacrifices of their families and striving to bring pride to their family's legacy, while for others the pressure was rooted in practical implications, such as taking on more debt if they failed a course or could not graduate on time. For example, one participant highlighted the perceived and real impacts of failing: *“I mean, [what] you're dealing with in the moment feels like [a] high-pressure situation. Like they are perceived, but they're also very real. If I fail my qualifying, it's an extra year of work, an extra year of school which equates to $70,000 that I just lost. If I fail my defense, it's an extra year or an extra semester of school, and that's not even exaggerating.”*--P28 (Latinx, Male).

Another participant described the pressure she placed on herself rooted in the desire to become a role model for others to look up to, and to bring pride to her family and community: *“I put that pressure on myself just because I wanted to be a good student in their eyes. I know the sacrifices they made from a young age to be in this country, and so I took it upon myself to really be a role model for my younger siblings, and a role model for my culture, for my people, for Latinos everywhere.”--*P09 (Latinx, Female).

Several participants also acknowledged the difficulty of asking for help, feeling that they were on their own to complete their degrees. A few participants explicitly mentioned experiencing imposter syndrome, thinking they were less qualified than their peers, especially peers who had already completed advanced degrees (such as in medicine), leading to feelings of inferiority and isolation. One participant reflected:

*“Part of it was just constant feelings of imposter syndrome which I think happens often with first-generation students. I experienced that feeling a little bit less in undergrad because there were so many more people who I felt were in the same boat as me. Compared to my graduate program, where … people had already graduated from medical school, and were here to also learn about public health. And that's great…but it was like, so this person has experienced this, and their parents are doctors.”* – P17 (Black, Female)

This quote demonstrates that participants were not only aware of their own lack of experience, but also of generational gaps in resources and knowledge relevant to graduate education that their families could not provide. In fact, participants recognize the imbalanced dynamics of power and privilege within their training experiences, but often felt they were unable to address structural disparities without institutional support. Individuals who were able to engage in institution-led initiatives described experiencing more agency to raise concerns through official channels. For instance, one individual described joining her institution's curriculum committee and felt that the experience provided her a platform to voice her concerns with her graduate education. These findings highlight the need for academic institutions to create more structured opportunities to partner with FGSOC in addressing structural barriers.

### 3.5 Theme 5: coping strategies for well-being

This theme captured how participants managed stress during their graduate training, often by leveraging opportunities to improve access to personal, social, and economic resources. Subthemes highlight the positive effects of supportive relationships, access to health-promoting resources, and the health benefits of alleviating financial burdens.

#### 3.5.1 Subtheme 5.1: benefits of a sense of belonging

Eight (32 %) participants described the positive impact of strong friendships, demonstrating that shared experiences provided motivation and reminded participants that they were not alone. One participant recalled: *“Motivating myself to keep going because they [classmates] were going through the same thing. I remember one of them, we were having the same classes at the same time. So, finding comfort in knowing that you're facing the same challenges, and you try to help each other as best as you can.”*--P24 (Latinx, Male). This quote highlights the importance of friendships in offering mental and emotional support, helping FGSOC navigate institutional structures and “hidden curricula” of their graduate education together.

Relationships with peers also promoted well-being by encouraging healthy habits through mutual encouragement. One participant described, “*My roommates were very much into health and running and making sure we were taking care of ourselves. I think that also kept us all motivated. And they were also all students at the School of Public Health. So yeah, that really helped us in that sense*.”--P06 (Latinx, Female). In addition to maintaining healthy habits, connecting with other first-generation students through culturally shared experiences, food, movies, and music, provided comfort and community.

On the other hand, participants also reported stress when belonging was challenged, in instances of disconnect or isolation based on aspects of identity. One participant described the psychological and emotional impact of facing classism: “*I had to deviate the conversation so that I didn't out myself as somebody who grew up low income. In some classes I had to protect myself from revealing my identity… and it was hurtful to be in a position like that, and in an institution that has a 5-year diversity, equity, and inclusion plan. I feel like they were learning how to implement this at a cost to my wellbeing.”*--P27 (Native American, Non-binary).

#### 3.5.2 Subtheme 5.2: accessibility to health-promoting resources

Six (24 %) individuals described accessible health-promoting resources as critical for maintaining healthy habits during their graduate education. Those who lived on campus described easier access to certain resources such as the campus gym. One participant described the benefits:

*“Since I was living on campus it was very different, amenities on campus were much more accessible. The cafeteria if I really needed like food in a pinch, or, most importantly, the gym that was like pretty much two blocks away from my residence. I was able to actually hone in on my physical health during grad school, because I used it as an outlet, for the stress of being in class and then having all the assignments due.” —*P18 (Asian, Male)

On the other hand, participants living off campus described facing structural challenges to access, such as lack of healthy food options in their neighborhoods, leading to decisions prioritizing convenience over healthier choices. These findings highlight the need to consider how graduate students' environments impact health behaviors and the importance of creating access to resources for those living off campus, which is often the case for most graduate students.

Access to food options is also tied to financial stability. Some participants described periods of food insecurity during their graduate training, lasting from a week to months while participants were looking for jobs or additional funding. This was often a result of a lack of a financial buffer to fall back on. Participants described weight loss, stress, and an urgent sense of survival as a result of periods of food insecurity. One international student shared: “*It was really hard and very difficult circumstances for me. I used to go to bed thinking where my next meal is coming from, how am I gonna even survive? Am I gonna graduate? What should I do*?”--P22 (Black, Male).

Campus food pantries provided a partial safety net to offset costs for students to address periods of food insecurity, but experiences varied depending on available options and food preferences. One participant recounted: *“I definitely didn't have enough money for groceries. There was a food pantry that was kind of like a hit or miss. I wasn't always trying to like eat potatoes and kale. I was grateful for what we had, but just some of the items weren't the best, but that was still an important resource to have.”*--P27 (Native, Non-binary). These findings highlight how environmental conditions and socio-economic factors impact access to health-promoting options for graduate students.

#### 3.5.3 Subtheme 5.3: alleviating financial burdens reduces stress

Nine participants (36 %) described experiencing relief when accessing financial support through jobs, scholarships, and paid internship. Financial stress was also raised as a byproduct of a lack of access to financial literacy, including management of credit cards and student loans. One participant highlighted the need for financial education:

“*At 19, you're not thinking about how these student loans [are] going to impact me when I'm 25, 26, 27? You need a group, whether it is other students, a department at an institution, or something that provides information about the consequences later on. Or even if it's like using your credit card, a lot of people don't have the guidance around using a credit card. From a financial literacy perspective I feel like that is something that is very much lacking, especially for people who don't have someone that they can fall back on for advice or guidance, like first gen students*.” —P05 (Latinx, Female)

Without financial assistance, participants described being in survival mode when managing daily expenses and debt:

*“It was really helpful to learn about scholarship opportunities and grants that students could apply for [to] fund their education. It reduced my stress in trying to figure out what's next. Am I gonna have to take out a loan? Am I gonna have to pay out of pocket? I was really grateful for that.”*--P07 (Black, Female)

Participants also identified other strategies to alleviate financial costs, such as living with parents to save on housing costs, which also helped to manage financial stress. For participants in vulnerable situations, such as undocumented students, relying solely on personal and familial funds to cover tuition created added stress and feelings of helplessness, compounding multiple stressors (pressure to achieve, financial instability, imposter syndrome). These experiences raise the need for more research into the varied structural barriers affecting FGSOC pursuing graduate training.

## 4 Discussion

This qualitative study investigated the perceived health and experiences of FGOSC in their pursuit of public health graduate training. Five key themes were developed through a reflexive analysis approach: (1) lived experiences facilitate FGSOC interest in public health, (2) challenge of navigating structural barriers of academic institutions, (3) interplay of support to meet needs and complete graduate education, (4) resource strains as stressors, and (5) coping strategies for well-being. The main themes were developed through analysis using Conservation of Resources (COR) theory with an intersectional approach to investigate how FGSOC navigated compounded structural and individual stressors. Furthermore, this study contributes to the limited body of literature that exists on the topic to highlight the value of lived experience of FGSOC as assets to enrich public health, the structural barriers FGSOC must confront, coping strategies adopted in their graduate training, and the perceived impact of these experiences to their health and well-being.

First, our findings reinforce recommendations from the Office of the Assistant Secretary for Planning and Evaluation to engage and involve individuals with lived experience to improve federal research, policy, and practice [42]. As public health aims to strengthen population health outcomes through diversifying and training on topics of structural racism and social justice, the lived experiences and personal connections of FGSOC to health systems will be an important asset to build on and learn from [43,44]. Participants identified how formative memories and experiences engaging with healthcare institutions provided insight into systemic gaps, motivating them to pursue public health degrees and contribute to positive impact. The idea that FGSOCs' intersectional lived experiences and cultural knowledge can add value to academia also subverts existing power structures and epistemic norms rooted in Eurocentric ideologies within higher education [45]. Embracing such perspectives creates opportunities to integrate lived experiences into curricula, practices, and institutional policies, thereby redefining academic research and practice to advance more equitable and culturally relevant models.

Our study results also corroborate findings of challenges that graduate students of color face, which include experiences of racism, isolation, lack of integration, mental health stress, and limited mentoring during their training [46]. Higher education is historically built on White institutional values [47], perpetuating forms of structural oppression through a legacy of decentering and erasing marginalized identities [48]. Participants described experiencing racism and microaggressions during their graduate studies, of feeling like “outsiders on the inside” or experiencing social or cultural disconnect from some peers, staff, and faculty [49]. Applying an intersectional lens allows for recognition that these experiences are shaped by multiple overlapping identities shaping compounding vulnerabilities within higher education settings, with implications on one's management of key personal, social, and economic resources.

From our analysis, there are clear opportunities for academic institutions to address the cultural and racial dissonance embedded in academia [50]. Participants identified the ongoing challenge of navigating “hidden curricula” during their graduate training and the impacts to their health, financial stability, and career directions. Therefore, proactively making dynamics of power and privilege transparent, and openly discussing aspects of the “hidden curricula” can reduce the isolating effects of structural barriers. Offering courses and academic spaces that support diverse perspectives beyond a Eurocentric/Western focus, and integrating decolonial praxis and critical approaches, such as critical race theory, to analyze systemic oppression can further empower students' contributions to structural change [[50], [51], [52]].

The study also suggests that financial fragility and the tension of financial stability are important to FGSOC. While financial challenges faced by graduate students generally have been documented, more nuanced dynamics across race/ethnicity and intersecting identities are understudied [53]. A majority of our study sample worked during their graduate studies and described the challenges of intermittent access to funding with short-term semester or summer jobs, creating conditions necessitating student loans with long-term financial consequences. Unpaid internships and the burdens of student loan debt also impact career options and create inequitable learning opportunities for students who are unable to take on financial risks [54,55].

Financial instability can also be seen as straining access to personal, social, and economic resources, creating stressors that undermine one's health. A recent study of U.S doctoral students (PhD) in psychology programs found that financial stress was associated with more time spent worrying about debt, more symptoms of depression and anxiety, and less sleep [56]. More research and documentation of the financial realities of graduate-level public health students is needed, especially for those of intersecting marginalized identities, such as international and undocumented students, who may face added structural barriers. Furthermore, no participants from the study mentioned accessing any form of financial literacy education or training during their graduate studies- interventions that some U.S academic institutions are implementing to support graduate student populations [53].

Our findings also suggest that fostering a sense of belonging is an important factor shaping the graduate education of FGSOC. Sense of belonging is defined “as a student's feelings about fitting in with peers within a particular community”, with decreased belonging associated with poorer grades and increased risk of not completing a degree [57]. Participants described the value of cultivating friendships that fostered belonging and provided emotional and academic support to fill institutional gaps. From an intersectional perspective, belonging is shaped by the intersecting identities of students, serving as an important social resource buffering stress and supporting coping strategies. Therefore, academic institutions can be more proactive in facilitating relationship building among students, advisors, faculty, and staff, while fostering a culture invested in equitable financial and social support for all students [21].

This study also highlighted the experience of food insecurity among some graduate students. While only a subgroup of individuals reported experiencing short-term or long-term food insecurity, more research into these experiences is paramount. A 2019 systematic review found a weighted mean prevalence of food insecurity of 50.9 % among undergraduate and graduate students [58]. A study of graduate students (those enrolled in a master's or doctoral programs) found 40.3 % of participants reported experiencing low to very low food security, with poorer mental health among those facing very low food security (high food insecurity) [59]. From a COR perspective, food insecurity represents a critical strain on financial resources, exacerbating stress and adversely influencing health. FGSOC of intersecting marginalized identities may be at greater risk to experience such resource strains and challenges.

Findings also reveal distinct experiences among master's-level students. Some master's-level participants reporting greater familial responsibilities as compared to doctorate-level participants. According to a recent 2024 graduate student profile, 35.5 % of master's-level students have dependent children, compared to 28.4 % of doctoral students [60]. Although limited demographic data exists for graduate students, parenting undergraduate students were more likely to identify as a person of color than as White [61]. Master's-level participants also emphasized the importance of securing employment during their studies, particularly positions offering tuition remission, reflecting the self-funded nature of most master's-level programs as compared to the financial support available for most doctoral programs [62]. Even among master's-level participants who received fellowships or scholarships to cover tuition, many still felt compelled to work to meet basic living expenses. Therefore, from a COR perspective, master's-level public health graduate students may face distinct financial and personal stressors and impacts on health during their training.

Across all graduate levels, participants emphasized the importance of mentoring for navigating academic and professional decisions. Specifically, doctoral-level participants highlighted how mentors supported them in managing personal challenges within academia, including imposter syndrome, racialized stress, and experiences of microaggressions and discrimination. Literature focused on graduate students of color has documented the exposure to racism, microaggressions, and systemic discrimination as linked to increased isolation and poorer mental health outcomes [46,63]. In response, scholars emphasize the need for culturally responsive mentoring to better support students “navigating marginality” of multiple intersecting identities. Culturally responsive mentoring prioritizes “a critical consciousness, decoding the hidden curriculum, and developing cultural awareness as mentors and mentees” to foster meaningful relationships that facilitate the recruitment and retention of diverse public health students and professionals, often best achieved through learning from the experiences of diverse faculty [64].

Our results also align with studies from other health disciplines, including medicine, pharmacy, dentistry, and nursing. Research in medical training has emphasized the need to address academic, financial, emotional, mentorship, and professional development barriers faced by FG medical students [65]. In pharmacy, financial concerns and perceived academic limitations have been identified as major challenges faced by FG students, and in dentistry, institutions recognized the necessity of making systemic policy changes to improve diversity in dental education programs [66,67]. Additionally, research from nursing education highlighted that FG PhD applicants were more likely to be rejected, with active faculty-applicant mentoring playing a key role in successful applications [68]. These existing studies recognize that FG students across health disciplines face structural barriers impacting personal, social, and economic resources. Public health is no different and can only benefit from more research exploring the experiences, factors, and implications for the training and well-being of FG students, especially FGSOC.

Lastly, our study explored perceived health experiences of FGSOC during their public health training. Participants described the impact of stress, isolation, and identity-related pressures on their perceived mental health. Furthermore, participants' health was influenced by familial responsibilities and stressors experienced during the COVID-19 pandemic. Our study found that despite struggling with health difficulties while managing resources during their training, they also made efforts to generate and protect resources, by sharing resources and information with peers, finding outlets for stress, and building meaningful connections with mentors, peers, friends, or family members.

This research has some limitations. While the sample size and makeup are appropriate for this study's methodological approach, the findings are not generalizable. First, we faced sampling constraints in our inclusion of Asian and Multiracial, FG, male participants to fulfill quota sampling. Furthermore, our study sample was predominantly U.S centric, with only a few international students and individuals with undocumented status. Since citizenship status was not a primary focus of this study, future research is needed to more fully examine the impact of these experiences. There are also future opportunities to expand research beyond binary gender categories to explore the experiences of non-binary identities. Lastly, validation strategies such as member checking or data triangulation were not employed due to resource limitations, which may have strengthened study credibility. Despite these limitations, the study contributes to the limited literature by providing initial data and findings into the experience of FGSOC pursuing graduate degrees in public health.

Overall, our findings highlight several new areas of learning in the field- specifically around identifying the assets that FGSOC bring to deepen public health's capacity to serve diverse populations, as well as highlighting the structural barriers faced by FGSOC in completing their graduate training. As one of the few studies directly drawing on the experiences of graduate-level FGSOC in public health, these findings underscore the distinct experiences, strengths, and challenges that this population face, as well as the opportunities for training institutions to more deeply engage and support these students. Looking ahead, future studies and programs should delve further into the intersectional experiences of graduate students in public health, focusing on more granular identities of ability, citizenship, sexual orientation, and gender to explore unique lived experiences and identify structural gaps to inform best practices, policies, and interventions for institutional change.

## 5 Conclusions/recommendations

This study is one of the few qualitative investigations into the experiences of FGSOC in public health graduate training programs. Our findings identify several key factors influencing FGSOCs' ability to complete their graduate public health training. These include drawing on lived experiences with health disparities and inequities as motivation for pursuing their degrees, leveraging support from networks and mentors when navigating hidden curricula and financial barriers, developing coping strategies to maintain well-being while facing resource constraints, and accessing health promoting resources. Our study extends existing research on the experiences of graduate students of color by centering the experiences of FGSOC. Based on these results, we also identify opportunities for programs to address structural barriers and implement systemic changes to improve student experiences and outcomes. The findings inform several recommendations for public health graduate programs, addressing institutional, academic, social, financial, and health-related realities faced by FGSOC. Our recommendations are summarized in Table 3, outlining concrete strategies that institutions can implement to enhance the success of FGSOC in public health graduate education contexts.

**Table 3:** Recommendations based on research findings.

Domains	Recommendations
Institutional Proactivity, Accountability, & Investment	• Foster regular outreach and relationship-building between staff, faculty, and FGSOC.
	• Utilize culturally responsive mentoring to support students' navigation of systems of higher education [64]. *All graduate programs*: Having mentors and advisors who can guide doctoral students of color and bridge cultural isolation is critical for student success [63,69].
	• Create a learning environment that values and integrates students' lived experiences into the curriculum and program [42].
Financial Education & Transparency	• Support and encourage students to access diverse types of fundings (departmental, external, institutional, etc.…) [53]. *Master's level programs*: Since most master's-level training is self-funded, identifying alternative sources for students to cover costs can alleviate financial stress.
	• Improve transparency around graduate funding opportunities and costs of living, including financial aid, assistantships, fellowships, and scholarships to clarify expectations and financial commitments [53].
	• Develop financial education programs for graduate-level students to explore the effects of student loans or other graduate studies related costs on long term financial goals. This can be achieved through partnering with other institutions to share resources, including on-demand online education around financing graduate studies (ie: Indiana University) [53,70].
Career Development & Workforce Readiness	• Assess evolving workforce needs and support upskilling/reskilling for public health students. Conducting job postings analysis may help to identify the skills needed by employers [55].
	• Develop partnerships with governmental institutions to facilitate internship and employment opportunities (ie: Academic Health Departments). Share resources and support students to apply for local, state, or federal government positions that require navigating additional systems [71]. *All graduate programs*: Public health graduates may be interested in pursuing governmental positions, many barriers (ie: burdensome application process, process taking too long) dissuade individuals from securing employment [72]. • Encourage exploration of lesser known career paths that leverage public health skills and competencies [73]. These roles may include occupational safety and health and addressing environmental hazards.
Accessible Mental Health Resources	• Ensure accessible, culturally appropriate mental health services and transparent policies and practices for graduate student well-being [74]. For instance, a participant mentioned their institution offering group therapy for FGSOC pursuing graduate degrees in health fields.
	• Foster supportive cohort spaces and peer connections, partnering with student organizations to enhance well-being [65,74]. *All graduate programs*: Cultivating these spaces can help to address isolation and to identify different support systems to navigate unfamiliar academic environments and systems within higher education.
Food security	• Strengthening partnerships or innovative collaborations to create healthy food options and access to physical activity for students living both on and off campus. Partnering with local gyms or grocery stores to offer student discounts, etc.…
	• Collaborate with students and staff to develop programs that assess and address food insecurity, including access to resources like food pantries [75].

## Availability of data and materials

The dataset created and/or analyzed during the current study is available from the corresponding author upon reasonable request.

## CRediT authorship contribution statement

**Kimberly Wu:** Writing – review & editing, Writing – original draft, Visualization, Software, Investigation, Funding acquisition, Formal analysis, Data curation, Conceptualization. **Sunshine Best:** Writing – review & editing, Formal analysis, Data curation. **W. Marcus Lambert:** Writing – review & editing. **Shokufeh Ramirez:** Writing – review & editing. **Christine M. Arcari:** Writing – review & editing. **Katherine P. Theall:** Writing – review & editing, Conceptualization. **Dovile Vilda:** Writing – review & editing, Writing – original draft, Supervision, Formal analysis, Conceptualization.

## Ethics approval

Ethics approval and consent to participate: This study was approved by the Tulane University Health Sciences Center Institutional Review Board (2023–535).

## Funding sources

This work was supported by the 10.13039/100006545National Institute on Minority Health and Health Disparities (NIMHD), Minority Health and Health Disparities Research Training Program administered by 10.13039/100005622Tulane ARMHR: Advancing Representation in Minority Health Research [award number T37MD001424].

## Declaration of competing interest

The authors declare that they have no competing interests.
